# Bacteriology

**Published:** 1904-10-08

**Authors:** 


### Progress in Medicine and Surgery.

### BACTERIOLOGY.

**Dysentery.**—Firth[1] regards the bacilli of dysentery as a specific group, representative of which are the organisms associated with the names of Shiga, Vaillard, and Kruse. Dysentery bacilli may be separated from other bacteria of the bowel which they resemble by the fact that they are pathogenic to guinea-pigs and by the fact that they give an agglutination reaction when tested with the blood of animals into whom the specific bacilli have been injected. The blood of animals so treated shows no antitoxic action. If the specific organisms are given to rabbits by the mouth or are injected into the bowel no result is obtained, but subcutaneous injection of cultures gives rise to dysenteric symptoms with ulcerating and sloughing areas in the cæcum. These symptoms could also be produced by the injection of dead cultures or of a sterile alkaline broth filtrate. The dysentery toxin appears to have a selective affinity for the large bowel and cæcum. Dysentery bacilli in desiccated rags and soil survived for 21 days, and at 22° C. they survived in water for 55 days. Spread on ordinary bread the bacilli survived for a week. The well-known fact that dysentery bacilli although constantly found in the mesenteric glands are yet never found in the blood, spleen, or other organs, leads us to classify the disease as a toxæmia with a localised focus of disease. Dysentery toxin was obtained by Todd[2] by prolonged growth of cultures in strongly alkaline broth. The toxin is a stable body, not destroyed by heating at 70° C. for an hour. It excites dysenteric symptoms in rabbits, and is often rapidly fatal. Todd succeeded in immunising a goat and a horse by means of increasingly large doses of the toxin. The serum of these animals was shown to have high agglutinative value when tested with B. dysenteriæ (Kruse). The serum also had antitoxic value but no anti-bacterial effect. The combination of the toxin and antitoxin in vitro does not take place immediately, but requires a certain time, and the rate of combination depends upon the temperature.

**Rabies.**—Negri about twelve months ago described a specific organism of rabies, a round or oval, elongated, pear-shaped body constantly occurring in the nerve cells and ganglia of the central nervous system. Their length was from 1 $\mu$ to 27 $\mu$ and their breadth from 1 $\mu$ to 5 $\mu$. These bodies he regarded as belonging to the protózoa. They can be detected in the fresh state or may be stained with eosin-methyl-blue. Amongst others who have confirmed this result is Luzzani[3] who has made a careful histological examination of the tissues of a boy aged 10 years who died from the effect of a dog-bite on the cheek. The parasites described by Negri were found in the brain cells of the cortex, in the cerebellum, the ganglion of the vagus, and in the sympathetic ganglia. The case was one of very short duration and the author thinks that this may account for the small size of the parasites which never exceeded 7 $\mu$ in length. Negri noted in dogs that in the furious form of the disease the parasites were most numerous in the brain, whilst in the paralytic form they were most abundant in the spinal ganglia and medulla.

**Notes on Bacteria.**—Rogozinski[4] has found that the lacteals of mesenteric glands of healthy animals may contain organisms such as B. coli, B. mesentericus vulgatus, B. subtilis, and cocci. Distinctive organisms such as B. prodigiosus, when fed to animals, could be readily detected in the lacteals and mesenteric glands, but not in the blood, liver, or spleen. Kohlhagge, it will be remembered, maintained that the empty human small intestine was sterile, and this finding is apparently confirmed by the observation of Rodella,[5] who examined the fluid in the hernial sac of five cases of strangulated hernia, and in all found it was sterile. Bastian[6] still maintains that organisms may arise *de novo* by heterogenesis. He summarises the work of the numerous observers who have found organisms in human tissues after death, and negatives the view that these organisms could exist in the tissues during life. The bactericidal properties of the blood would, he says, militate against their existence, and the well-known facts of modern surgery point to the aseptic nature of the body tissues during life. Again, the majority of bacterial forms found in tissues after death are non-mobile and would have no power of migrating through the gut wall. Bastian believes that the short motionless bodies first found afterwards develop into long bacillary mobile forms. In this last point he is supported by Nakanishi[7] who finds all bacteria in their young state are short uninucleated cells, the membrane of which is thin, smooth, and structureless. Outside this membrane there may be a gelatinous deposit, but this is an excretory product. The cell mass is made up of two layers of cytoplasm surrounding a round or oval deeply staining nucleus. Spore formation is an intracellular encapsulation of the nucleus and the neighbouring cytoplasm. These details of structure he has made out by means of special stains made by dissolving aniline dyes in ascitic fluid. The action of radium on micro organisms has lately been studied by Green.[8] He finds that there is a marked germicidal action if the radium be in close contact with the organisms, but that as the distance is increased this power is lessened. At a distance of from 1 to 2 mm. pyogenic cocci and active organisms in calf lymph were destroyed in from 10 to 22 hours. Spore-bearing organisms were more resistant, and were not killed by an exposure of less than 72 hours. At a distance of 10 centimetres no bactericidal action could be detected. After a long exposure to radium bacteria show signs of radio-activity and affect a photographic plate, and may give off photo-actinic rays for a subsequent period of three months. Jeannin[9] has investigated the bacteria found in the mouth of the infant and suckling child. The mouth is sterile at birth, but bacteria are to be found in it within a few hours, even if the child has not been put to the breast: these forms include the streptococcus salivæ, streptococcus ærobius micros, and staphylococci, and the number of species is rapidly increased after the child has once been suckled. Anærobic forms are the more numerous, and bacteria are more common in the mouths of sickly than of healthy children.

Streptothrix Infection.—It is now generally conceded that the actinomycosis should be classed amongst the streptothrix group and it might well be described as the streptothrix bovis communis, as opposed to that rarer disease caused by the streptothrix of Eppinger. Only five cases of this latter condition have been recorded, the lesions occurring chiefly in the brain or bronchial glands. To these cases a sixth is added by M'Donald.[10] The patient, a woman aged 65, died from symptoms of pleurisy and pyæmia. Post-mortem examination showed lesions in the brain, lungs, pleura, and kidney, resembling those of caseous tubercle. From these lesions a pure growth of streptothrix was obtained. On agar the growth first appears as a whitish-yellow film which assumes a wrinkled appearance and which is firmly adherent to the medium. Gelatine is not liquefied and broth shows a dense, white, felted film with stalactite projections. In milk and on potato the growth is orange-tinted. This growth on potato is seen to consist of a tangled mass of filaments, branching dichotomously, and bearing aerial hyphæ with chains of spores. Stained by Gram's method these may appear either uniform rods or coccal-like chains. Old cultures stained by Zeehl-Neilsen's method show acid-fast properties. The cultures were pathogenic to guinea-pigs and rabbits and produced characteristic tubercle-like nodules. Borelius[11] reports three cases of circumscribed mobile nodules due to actinomycosis occurring in persons suffering from symptoms of mechanical irritation of the bowel with occasional rigors and fever. The ray fungus was readily detected in the fibrous hard tumours removed by operation.

[1] Brit. Med. Jour., March 19, 1904. [2] Ibid, Dec. 3, 1903. [3] Gaz. Med. Ital., Feb. 18, 1904. [4] Ver. der Natur. d'Akad. im Krakow, Bd. xlii. [5] Rit. Med., Nov. 18, 1903. [6] Lancet, July 19, 1904. [7] Centrab. f. Bakt., Bd. xxx., No. 3, 4, 5, 6. [8] Proc. Rey. Soc., May 5, 1904. [9] L'Obstétrique, July 1904. [10] Scot. Med. and Surg. Jour., April 1904. [11] Nord. Med. Arkiv. Afd. I., No. 6, 1903.

